# Perceived importance of recruitment and retention measures for bachelor-qualified nurses in German hospitals: mixed-methods study

**DOI:** 10.1136/bmjopen-2025-113495

**Published:** 2026-08-10

**Authors:** Claudia B Maier, Charlotte Reidt, Julia Köppen, Mandy Baumann, Ana Mazur

**Affiliations:** 1 School of Public Health, Bielefeld University, Bielefeld, Germany; 2 Department of Healthcare Management, Technische Universität Berlin, Berlin, Germany

**Keywords:** Nurses, Nursing research, Nursing Care

## Abstract

**Abstract:**

**Objectives:**

Hospitals across Europe are under pressure to recruit and retain a well-qualified nursing workforce amid ongoing shortages. The study aimed to assess the perspective of bachelor-qualified (BSN) nurses compared with vocationally educated nurses on the relative importance of various recruitment and retention (R&R) measures to determine tailored, suitable R&R measures according to BSN-specific needs.

**Design:**

A convergent mixed-methods study combining a survey and semi-structured interviews through triangulation, conducted as part of the BSN4Hospital study.

**Setting and participants:**

BSN and 3-year vocationally educated nurses were surveyed in 22 German hospitals with R&R strategies and above-average numbers of BSN. In addition, interviews took place with BSN, chief nursing officers (CNO) and nurse scientists and those working in strategic units (NSC/other).

**Measurement:**

The survey included items related to R&R (work environment, onboarding, salary, shift work, work-life-balance, leadership, roles, tasks), perceived importance and the top three R&R measures. Interviews focused on targeted R&R measures, implementation and challenges.

**Results:**

A total of 1856 nurses participated in the survey (282 BSN and 1574 vocationally educated nurses). Semi-structured interviews were conducted with 19 individuals (6 BSN, 8 CNO and 5 NSC/other). On recruitment measures, a significantly higher proportion of BSN (82.6% vs 52.7% of vocationally educated nurses, p<0.001) rated salary according to nurses’ qualifications as highly important. Good teamwork and work-life-balance were valued similarly by both groups. Additional recruitment measures emanating from the interviews were BSN-specific onboarding, mentorships, targeted job advertisements outlining BSN profiles, hospital networking and external communication strategies. On retention, in addition to salary, a higher proportion of BSN (63.8% vs 40.1%, p<0.001) highly rated individual career opportunities, advanced clinical tasks (59.9% vs 32.0%, p<0.001), independence (55.3% vs 47.8%, p=0.020) and practice development projects (47.5% vs 20.0%, p<0.001). The interviews underlined the importance of clinical career opportunities, expanded roles and protected time for additional tasks. Multiple measures were identified as relevant for both R&R.

**Conclusion:**

Several R&R measures specific for BSN were identified suggesting that a tailored approach is needed. An integrated management approach that recognises BSN qualifications can support workforce stability and high-quality patient care.

**STRENGTHS AND LIMITATIONS OF THIS STUDY**

- This study used a mixed-methods design on recruitment and retention (R&R) by nursing education and focused explicitly on bachelor-qualified nurses (BSN), allowing for an in-depth, nuanced understanding of BSN-specific needs; to our knowledge, it is the first study of its kind.
- The mixed-methods design enabled triangulation of quantitative and qualitative data, which was instrumental in contextualising findings and identifying unexpected factors.
- The survey focused on innovative hospitals and was based on a large quantitative sample, highlighting established and innovative R&R measures that may serve as examples for others.
- The interviews included the views of practicing BSN as well as nursing management and nurse scientists which was a strength; however, the number of BSN interviews was small and nurses with vocational education were not interviewed.
- The focus on hospitals with established and innovative R&R measures limits the generalisability of findings to less experienced organisations, but suggests options for improving R&R.

## Introduction

 Many countries in Europe and globally are facing a nursing shortage, which pre-dated the COVID-19 pandemic and aggravated thereafter.^1 2^ Reasons are multifold and include low intake into nursing education, high staff turnover and attrition rates among clinical nurses, influenced by unfavourable work environments, high workloads, limited career possibilities and decision-making, among others.^3 4^ Hence, there is a high need to identify effective and sustainable recruitment and retention (R&R) measures. A review of several case studies on the R&R of health professionals across Europe suggested bundles of measures instead of single interventions,^5^ including professional and personal support, strong interdisciplinary teamwork, career development and effective leadership. A systematic review of 23 studies showed that positive leadership, characterised by participative decision-making, an appreciative attitude towards nurses and a supportive work environment fostering team cohesion, was significantly correlated with nurses’ intentions to stay in their positions.^6^ Adequate salary and high job security through permanent employment contracts have also shown to be relevant factors.^7^ Additional measures to promote the retention of newly qualified nurses in clinical care and reduce their turnover encompass internship or trainee programmes, as well as orientation and transition-to-practice initiatives incorporating mentoring as part of the onboarding process.^8^

While there have been several studies analysing R&R strategies specifically for nurses,^5 6 9 10^ few studies have analysed effective R&R strategies by nursing qualification levels. Yet, the nursing qualification and skill-mix has been diversifying across Europe and globally. The majority of countries in Europe have moved nursing education to higher educational institutions and established Bachelor of Science in Nursing (BSN) either as the sole entry to the profession or as an additional educational path to the traditional vocational education at nursing schools.^11 12^ International studies show that an increase in academic qualification in nursing and nurse staffing levels are associated with improvements in patient safety, higher quality of care and increased job satisfaction among nursing staff.^13–18^

Nursing education in Germany and other European countries has traditionally been based on a 3-year vocational programme, usually offered by nursing schools linked to hospitals. In Germany, for many years, this pathway constituted the primary route into the nursing profession. Although academic education at the bachelor level has been introduced in the 1990s as part of pilot programmes. Only as of 2020, bachelor education was formally established as a regular additional qualification pathway to nursing through the introduction of the Nursing Professions Act. Bachelor education takes 3.5–4 years and is offered at universities of applied science or universities.^19^

Both educational pathways lead to professional qualification and conclude with a state examination.^20^ Although each route qualifies nurses for the role as professional nurse, they differ in their skills and competencies with regard to scientific skills and methods, evidence-based practice, critical appraisal skills among others.^19^ These enhanced competencies were suggested as enabling BSN to respond to the growing complexity and rising demands of healthcare needs.^20^

Despite a threefold increase in the number of bachelor programmes in Germany from 2019 to 2021,^21^ the percentage of nurses with a minimum bachelor degree working in patient care has remained low. A 2024 study found that only 3.18% of nurses in university hospitals in direct patient care had a bachelor degree or higher, reflecting a low percentage compared with most other European countries.^22^ In a 2019 sample of 575 hospitals in Germany, the proportion of nurses with bachelor degree or higher among all nurses was 0.59% in total and 0.41% working in clinical care.^23^ In 2012, the German Science and Humanities Council recommended that 10–20% of nurses in training should be educated at the academic level, increasing the target to 20% in its 2023 update.^21 24^ In 2022, a study found that the percentage of nursing students in bachelor programmes was approximately 0.97% of all nurses undergoing primary nursing education, which has been far below the recommendations.^21 25^ In addition to increasing the number of bachelor programmes and graduates, attention has shifted towards recruiting and retaining BSN in clinical care. Therefore, it is essential to understand the factors that influence theR&R of this group.

In Germany, there has been limited evidence on BSN in clinical roles and practice settings.^23 26 27^ Challenges have been reported in identifying suitable areas of work in clinical practice and implementing organisational adjustments.^28^ A limited number of hospitals have established frameworks for skill or grade mix with job profiles tailored to each nursing qualification level.^23^ The lack of clearly defined tasks and job profiles by qualification has been referred to as one of the barriers to recruit, deploy and retain academically educated nurses.^26 29^ The German Federal Ministry of Health has acknowledged the need to align roles, tasks and competencies by nurse qualification and has drafted a new law to allow bachelor and higher qualified nurses certain expanded competencies and tasks.^30^ Furthermore, salary structures and tariffs have been shown to be used differently by hospitals which leads to a lack of transparency between the salary of academically and vocationally educated nurses. Overall, salary tariff structures in Germany depend primarily on the tasks and not solely on the level of qualification.^23 31 32^

Research on the R&R of nurses has predominantly focused on all nurses irrespective of qualification. It has largely neglected the views and perspectives of BSN, leaving a research gap in Germany and internationally. This is of particular relevance due to the increasing numbers of BSN in Europe and worldwide. The views of BSN on effective R&R in clinical care are critical for hospital management and policymaking to develop tailored strategies that support the retention for this group of nurses.

This study’s overall aim followed two interrelated objectives:

1. To assess and compare bachelor-qualified and vocationally educated nurses’ perceived importance of a set of recruitment and retention measures.
2. To identify which measures were particularly relevant for bachelor-qualified nurses and reasons thereof, drawing on in-depth views from BSN, management and scientists.

## Methods

The study BSN4Hospital (funded by the German Federal Ministry of Education and Research), employed a convergent mixed-methods design, with equal priority given to the quantitative (survey) and qualitative (interviews) arms, in accordance with the study protocol.^33^ A patient representative of a major organisation contributed to this study through participation in qualitative interviews. For this analysis, we focused on the identification of R&R measures based on the perspective of BSN in comparison with professional nurses with a 3-year vocational education from nursing schools, complemented with in-depth insights on specific R&R measures for BSN through the qualitative arm of the study. The study purposively recruited innovative hospitals in Germany, characterised by two elements: (1) an above-average proportion of nurses holding at least a bachelor’s degree working in direct patient care (>2% of university hospitals and 1% of non-university hospitals). The information was publicly available, retrieved from the ‘academic nursing degree’ specification of the 2020 nursing budgets submitted to the Institute for the Hospital Remuneration System (InEK)^34^ and (2) the adoption of specific, innovative R&R strategies, aimed at increasing or at minimum, retaining nurses with BSN qualification. The websites of hospitals with a higher than average proportion of BSN were searched for ‘innovative’ R&R measures at the organisational level, including interventions to recruit, employ or retain nurses with a bachelor’s degree. The information was obtained through hospitals’ mission statements or other strategic documents, as well as specific R&R interventions and programmes. The reporting of this study follows the GRAMMS checklist (Good Reporting of A Mixed Methods Study)^35^ (online supplemental, table 1) and the CROSS checklist (Checklist for Reporting Of Survey Studies)^36^ (online supplemental, table 2).

### Quantitative part of the study: survey

The survey was developed in 2022 in several rounds and piloted internally with researchers, the study’s advisory board; and externally with three nurses (no demographic or other data were collected) from one hospital which did not participate in the final survey.^33^ The survey items on recruitment (reasons for choosing a hospital) and retention (reasons for staying in a hospital, defined as for a minimum of 5 years) were developed based on the existing literature,^33^ covering 13 recruitment-related and 21 retention-related items. The recruitment-related themes comprised hospital and work environment, onboarding programmes, salary, contract, shift work and work-life balance. The 21 retention-related items addressed leadership, corporate culture, roles and clinical tasks, additional aspects on salary, contract and work-life balance. Some items were identified as relevant for both R&R and were therefore included in the analyses and assessed separately for each domain. Additionally, respondents were asked to select their top three R&R measures. Sociodemographic data as well as detailed information on qualifications were also part of the survey.

Data collection took place from June to September 2023 in 22 hospitals in Germany in three 4-week waves, scheduled to avoid regional vacation periods at the hospitals’ request. Hospital staff identified wards with at least one BSN and coordinated the distribution of the survey and data collection within the participating hospitals. Wards with no BSN were excluded. The survey was conducted in 21 hospitals as an online version only, except for one hospital which requested online plus paper-based surveys. All acute care wards in hospitals were included, except for psychiatry wards, as described in the protocol.^33^ All professional nurses over 18 years of age with a minimum of 3-year qualification were eligible to participate. Excluded were nursing assistants and non-nursing staff. Details on the sample size calculation are provided in the study protocol.^33^

The anonymous online survey was conducted via SoSciSurvey, for which specific URL as well as QR codes were created for each of the hospitals and communicated to the nurses via posters and flyers. In SoSciSurvey, IP addresses are by default not recorded. Informed consent was obtained from all participants electronically prior to the survey; participants were required to provide consent before accessing the questionnaire. Hospitals received weekly feedback on the number of participating nurses by qualification, as a means to keep up the motivation in hospitals and increase response rates. Response rates were calculated as the proportion of completed questionnaires among invited nurses in hospitals with available denominator data (20/22 hospitals).

The survey data were analysed descriptively, and group differences between BSN and 3-year vocationally educated nurses were examined using χ² tests. As the continuous variables age, years in the profession and years in the current hospital were not normally distributed, group differences were tested using the Mann-Whitney U test and results are reported as median (Q1–Q3). The significance level was set at p<0.05. The R&R items were measured using a four-point Likert scale and were dichotomised for analysis into ‘very important’ versus all other categories (‘not important’, ‘less important’, ‘important’). Due to pronounced ceiling effects when combining the categories ‘important’ and ‘very important’, analyses focused on responses rated as ‘very important’. In addition, the three highest-ranked R&R items, defined as those most frequently rated as ‘very important’, were analysed descriptively. Only complete cases were included in the analyses. As information on qualification (BSN, vocational training) was collected in the final section of the survey; participants with missing data on qualification were excluded. No weighting or other statistical adjustments were applied. All analyses were conducted using IBM SPSS Statistics, V.28.

### Qualitative part of the study: interviews

As part of the qualitative arm of the study, individual interviews were conducted following semi-structured guides developed for different target groups. The interview guides were developed iteratively within the research team and were informed by previous R&R studies on nurses and the current situation in Germany. Moreover, additional survey items (specific clinical tasks and roles) informed the development of the interview questions to ensure alignment between the qualitative and quantitative components of the study. For this analysis, in order to obtain in-depth insights on effective R&R measures, interviews with BSN working in patient care (to cover the perspective on clinical nursing), hospital chief nursing officers (CNO) (to cover the management perspective) and nurse scientists and those working in strategic units (NSC/other) (research/science perspective) were analysed. Written informed consent was obtained prior to each interview. The interview guide began with an introductory question, followed by six thematic sections (eg, implemented and recommended R&R strategies, measures specifically for BSN, roles and clinical tasks for BSN, salary, barriers, facilitators) and concluded with a final general question. Additionally, a short survey was developed to collect socio-demographic data.

### Sampling strategy

For the interviews, 7 of the 22 hospitals participating in the survey were selected through purposive sampling and gatekeeping, selecting a mix of university, teaching and non-teaching hospitals, larger and smaller (<500 beds) hospitals. In these hospitals, the CNO were directly contacted by the researchers and invited for an interview to obtain the top nursing management perspective. In addition, the hospitals identified suitable resource persons covering management, research as well as BSN working in direct patient care in these hospitals.

### Data collection and data analysis

Following the convergent-parallel mixed-methods design, interviews and survey data were collected simultaneously. The interviews took place between May and November 2023. Interviews were conducted face-to-face in the hospitals or online, by three researchers experienced with interviewing techniques (JK, JoK, SE). All interviewees filled in the short survey and the interviewers made field notes after each interview.

The interviews were transcribed verbatim by a transcription company in compliance with German data protection laws and anonymised to ensure confidentiality. Researchers verified each transcript against the audio recording for accuracy before analysis.

The qualitative data analysis was conducted by two researchers (MB and CR) with MAXQDA (V.24.4.1), following qualitative content analysis as described by Kuckartz and Rädiker,^37^ and applying a deductive-inductive approach. Deductive categories were derived from the interview guide and the survey, while additional inductive categories were developed from the interview data. All interviews were coded using the same coding framework. Interviews from the different professional groups were analysed as a single dataset, as they addressed a shared research focus. At the same time, participants’ professional roles were taken into account during coding and interpretation, to capture similarities and differences in perspectives across clinical, managerial and academic contexts.

To ensure rigour in the coding process, both researchers independently coded two transcripts each and compared their coding. The initial category system was then discussed, refined, and iteratively developed through repeated engagement with the data. Final categories and subcategories were defined and organised into a coding framework (online supplemental table 3). To enhance quality and coherence of the themes, define the thematic boundaries, and refine the analyses, additional discussions were held with two researchers (CBM and JK). The dataset was judged to be sufficiently rich to allow for meaningful coding and the development of themes relevant to the research question. Since the interviews were conducted in German, relevant quotes were translated into English and verified by an additional author for accuracy. Quotations were purposively selected to highlight key themes. While all interviews contributed to the analysis, quotations were selected to most clearly illustrate the identified themes, rather than reflect group proportions.

### Triangulation

Triangulation followed the convergent mixed-methods design using a parallel analysis approach. Quantitative and qualitative data were analysed independently and then integrated through systematic comparison. This process involved identifying similarities, differences and complementary findings across both datasets, supported by a triangulation matrix. The integrated analysis was used to contextualise and enrich the interpretation of the results.^35 38 39^

## Results

### Sample characteristics: survey

A total of 1856 nurses (77.5% female) participated in the survey, including 282 BSN and 1574 nurses with 3-year vocational education (table 1). The overall response rate was 14.0% (completed surveys). BSN were younger than vocationally educated nurses (BSN: median age 31 years (Q1–Q3 27–40) vs vocationally educated nurses: 37 years (29–49), p<0.001), and had fewer years of work experience, both in the current hospital and in the profession. They were also significantly less likely to have children (21.3% vs 30.3%, p=0.002). A higher proportion of BSN worked as nurse managers and was employed full-time compared with vocationally educated nurses. The majority of nurses worked in intensive care units, followed by surgery and internal medicine.

**Table 1:** Sample characteristics of survey respondents

	BSN	Vocationally educated nurses§	Total
	n=282	n=1574	N=1856
Female, %	76.6	77.7	77.5
Age (median, (Q1–Q3))	31 [27–40]**‡**	37 [29–49]**‡**	36 [29–48]
Years in current hospital (median, (Q1–Q3))	5 [3–10]**‡**	10 [4–21]**‡**	8 [4–20]
Years in profession (median, (Q1–Q3))	8 [5–15]**‡**	14 [7–25]**‡**	13 [6–25]
Children up to 17 years, %	21.3†	30.3†	28.9
Nurse manager, %	22.0‡	12.7‡	14.1
Full-time work, %	66.0‡	52.9‡	54.9
Temporary employment, %	3.9	2.2	2.5
Ward, %			
ICU	24.1*	30.4*	29.5
Surgery	14.5	14.8	14.8
Oncology	12.8	11.1	11.4
Internal medicine	12.4	11.8	11.9
Paediatrics, neonatology	8.5	10.5	10.2
Obstetrics	5.0	2.8	3.1
Emergency department	4.3†	1.8†	2.2
Neurology, stroke unit	3.5	4.0	3.9
Other	14.9	12.8	13.1

* p<0.05

† p<0.01

‡ p<0.001

§ Professional nurses with three3-year vocational education from nursing schools.

BSN, Bachelor of Science in Nursing; ICU, intensive care units; Q1, first quartile; Q3, third quartile.

### Sample characteristics: interviews

A total of 19 individual interviews were conducted (6 with BSN, 8 with CNO and 5 interviews with NSC/other). The recorded interviews had an average duration of 48 min for BSN, 65 min for CNO and 75 min for NSC/other. The average age of the interviewed BSN was 27.7 years, considerably younger than CNO and NSC/other (table 2). CNO had the most years of work experience, while NSC/other had worked the longest in their current position and all had a master’s degree. Neither CNO nor NSC/other were actively involved in clinical practice.

**Table 2:** Sample characteristics of interviewees

	BSN	CNO	NSC/other
	n=6	n=8	n=5
Age (mean, (SD))	27.7 (3.4)	57.6 (6.8)	48.4 (3.4)
Years in current position (mean, (SD))	2.0 (0.8)	8.9 (4.6)	9.6 (5.2)
Professional qualification (multiple responses possible)			
3-year vocational training	6	7	5
Bachelor’s degree	6	2	3
Master’s degree	0	3	5
Other nursing-related degree	0	4	2
Other non-nursing degree	0	1	0
Currently studying (yes)	2	2	1
Management and/or employee responsibility (yes)	3	8	3
Currently working in clinical practice (yes)	6	0	0
Years of work experience in direct care (mean, (SD))	4.0 (2.2)	15.1 (6.5)	7.6 (3.0)
Total work experience in healthcare (mean, (SD))	4.5 (2.9)	36.9 (6.5)	26.0 (2.8)

BSN, Bachelor of Science in Nursing; CNO, chief nursing officer; NSC, nurse scientist.

### Recruitment: quantitative findings

When asked about the importance of specific recruitment-related measures for choosing a hospital as a future employer, the highest proportion of all nurses, irrespective of their qualification, responded that good teamwork (BSN: 91.1%, vocationally educated nurses: 89.6%, not significant (n.s.)) and structured induction programmes for new staff (85.5% vs 86.6%, (n.s.)) were very important (table 3). Good teamwork was rated as one of the top three reasons for choosing a hospital in both groups. Work shadowing, modern workplace equipment and the reputation of a hospital were considered of lower importance.

**Table 3:** Recruitment measures: high importance and top three, by BSN and professional nurses with vocational education

	BSN n=282		Nurses with vocational education‡ n=1574		Total N=1856
‘Very important’	%	Top 3	%	Top 3	%
Hospital environment and onboarding					
Good teamwork	91.1	Yes	89.6	Yes	89.8
Induction programme for new staff	85.5		86.6		86.4
Possibility of work shadowing before signing a contract	56.0		55.1		55.2
Modern workplace equipment/digitalisation	56.0		56.1		56.1
Good reputation of the hospital	23.0		21.5		21.8
Salary and contract					
Salary according to level of professional qualification and training (specialisation, bachelor’s, master’s)	82.6†	Yes	52.7†		57.3
High job security (until retirement)	46.8*		54.3*		53.1
Single payment on signature of contract (eg, €4000 to €5000)	23.8*		30.9*		29.8
Shift work and work-life-balance					
Good compatibility of work and family/leisure time	81.6	Yes	82.1	Yes	82.0
Reliable duty rosters	81.6*		87.0*	Yes	86.2
Prompt compensation with extra time off after overtime/stressful shifts	64.5*		71.5*		70.5
Financial compensation after overtime/stressful shifts	50.0*		58.3*		57.0
Possibility to waive night shifts	28.0		33.5		32.7

* p<0.05.

† p<0.001.

‡ Nurses with 3-year vocational education from nursing schools.

BSN, Bachelor of Science in Nursing.

On salary and contractual factors, 82.6% of BSN (vs 52.7% of vocationally educated nurses, p<0.001) cited salary by qualification level as very important, which was one of the top three recruitment factors among BSN. This factor was the only item among all recruitment questions with a significantly higher proportion of BSN compared with vocationally educated nurses. Other salary or contractual features were of significantly less importance to BSN compared with vocationally educated nurses.

The interplay between shift work in relation to work-life-balance was considered very important to both BSN and vocationally educated nurses, although consistently of a higher proportion among vocationally educated nurses. There were significant differences between the two groups with regard to reliable duty rosters (81.6% vs 87.0%, p=0.014), extra time off after overtime or stressful shifts (64.5% vs 71.5%, p=0.018) and financial compensation after overtime/stressful shifts (50.0% vs 58.3, p=0.010).

### Recruitment: qualitative findings

Mirroring and expanding on the quantitative results, the interviews showed that (1) relevance of teamwork; (2) structured onboarding for BSN; (3) salary by qualification; (4) job advertisements with a BSN-specific profile and (5) hospital’s external communication strategies on BSN were considered as relevant measures.

### Relevance of teamwork for job decisions

Most interviewees considered some form of early contact with potential future teams on wards as highly relevant. Several of the interviewed BSN described that they had taken steps to gain first-hand insights into how potential future teams would collaborate. Examples included experiences during clinical training as part of the bachelor’s education or work shadowing. One BSN explained that the prospect of a good team was more important in her final decision than the preference for a specific specialty:

During practical training [as part of the Bachelor] you go through several departments and I thought cardiology was really great from a technical point of view and I thought gastroenterology, where I am now, and hematology were ok, but it wasn’t really an area that I had seen for myself. But the team was incredibly great and I always felt good in the team and could very well imagine working there, and then I realised that a good team structure, a stable team structure, is ultimately more important to me than the actual department I want to work in. (ID21, BSN, Pos. 32)

### Structured onboarding programmes

Onboarding or induction programmes for new staff, for example, trainee programmes for BSN, were seen as highly relevant by all BSN in establishing their new roles in clinical practice. One BSN noted the need for more individualism in trainee programmes. Having a mentor or tutor during the onboarding phase was considered an asset to support BSN individually during the transition phase from the student role to a BSN:

… and then … to create a transition so that you really leave your BSN studies and don’t end up in a phase where you lose motivation, but have someone directly, maybe a kind of tandem or contact person, where you can say ‘Yes, okay, that’s how it’s going now and I can turn to them, if I need someone directly’, so that there is someone at your side. (ID10, BSN, Pos. 90)

Most CNO and NSC/other highlighted that specific, structured onboarding programmes for BSN are essential, given considerable variations in qualifications and skills among BSN after graduation.

### Salary by qualification

All six interviewed BSN discussed payment by level of qualification as a crucial aspect of effective recruitment strategies. Five BSN mentioned the importance of salary according to level of qualification which they stated should be higher than that for nurses with vocational education. BSN were paid the same salary as vocationally educated nurses, which was seen as a strong demotivating factor.

And the financial aspect is also at the top of the list of attractiveness. Because in other jobs, the higher your education, the better you get paid. (ID21, BSN, Pos. 962)

Two BSN explicitly stressed this factor as highly relevant during the recruitment process.

So the first thing that frustrated me was actually that when I was hired, the sentence was said: ‘We do not pay for degrees.’ And that was the first frustrating moment. (ID21, BSN, Pos. 24)

CNO and NSC/other also acknowledged the importance of adequate salary levels based on qualifications. They also emphasised that salary is crucial for both R&R and should be considered together.

### Job advertisements with BSN-specific profile

All BSN stated that BSN-specific job advertisements by hospitals were essential for effective recruitment. Most BSN preferred specific job profiles aligned with their qualifications and specific roles that differentiate them from vocationally educated nurses. One BSN mentioned that the lack of role clarity in a previous job prompted the job search.

The hospital where I worked before tried to keep me… by saying: ‘We can look into developing tasks for you after your studies’, but it wasn’t clearly defined. That made me feel insecure. You didn’t know exactly what to expect and [the other hospital] advertised this position. And I read the job description and it was a perfect fit aligning with my BSN studies. (ID10, BSN, Pos. 14)

CNO and NSC/other also highlighted areas for improvement in role clarity, and emphasised the need to allocate time for scientific work, for example, through practice development projects.

### Hospital’s external communication strategies on BSN

Several interviewees mentioned that hospitals did not sufficiently communicate on their website or other media their willingness to employ BSN and BSN-supportive work environments. Three interviewees specifically described this lack of transparency as a barrier when searching for hospitals supportive of academic nursing and BSN integration. Communication with the public and networks or partnerships were also mentioned by many interviewees as an attractive recruitment strategy.

… And then, of course, if there are projects, I would try to advertise them to the public. So sometimes there are great things that you find in the workplace that you can take to the community, to cooperate with a university, to advertise there, where there are students, … in the vicinity*…* (ID14, CNO, Pos. 66)

In this context, BSN, CNO and NSC/other considered cooperation with local universities beneficial. This would provide nursing students and graduates the opportunity to familiarise with the hospital, while also allowing CNO to showcase their hospital to a larger pool of potential BSN and the wider public.

### Recruitment: triangulation

The synthesis of the survey and interview findings reinforced that BSN highly value teamwork, structured induction programmes (eg, onboarding) and salary based on qualification levels as important recruitment measures. The interviews provided additional themes that were not covered by the survey questions, such as the proactive job search of some BSN for a suitable position. Some BSN actively sought ways to assess teamwork before applying to a job. They also highlighted the importance of BSN-specific job advertisements with clear role descriptions for clinical practice. Additionally, BSN referred to the need for more public communication by hospitals on BSN and active networking with academic and other institutions.

### Retention: quantitative findings

Over 80% of all nurses irrespective of their qualification rated good nurse staffing levels and good teamwork as very important for retention, defined as the decision to stay in a hospital for 5 years or more (table 4). There was a significantly higher proportion of BSN (63.8%; vocationally educated nurses: 40.1%, p<0.001) which considered individual career opportunities as a very important retention factor.

**Table 4:** Retention measures: high importance and top three, by BSN and professional nurses with vocational education

	BSN n=282		Vocationally educated nurses§ n=1574		Total N=1856
**‘Very important’**	**%**	**Top 3**	**%**	**Top 3**	**%**
Hospital and work environment					
Good nurse staffing	86.5	Yes	88.9	Yes	88.6
Good teamwork	85.1		86.3		86.1
Modern workplace equipment/digitalisation	44.3		44.9		44.8
Leadership/corporate culture					
Good leadership skills of the supervisor	74.1		75.5		75.3
Noticeable recognition and appreciation from colleagues/supervisors	66.0		65.8		66.0
Career opportunities tailored to my personal goals	63.8**‡**		40.1‡		43.7
Flat hierarchies	45.2		44.0		44.2
Salary and contract					
Salary according to level of professional qualification and training (specialisation, bachelor’s, master’s)	77.0‡	Yes	46.6‡		51.2
Generally higher pay scale for all nursing staff	62.8†		74.1‡	Yes	72.4
Reduction in working hours with full pay compensation after 10 years of employment (eg, 35 hours per week)	58.9‡		70.5‡		68.8
Financial compensation after overtime or stressful shifts	42.6‡		53.9‡		52.2
High job security (until retirement)	40.8*		47.6*		46.6
Roles and tasks					
Advanced tasks for nurses (including advanced clinical assessments) by professional qualification (specialisation, bachelor’s, master’s)	59.9‡		32.0‡		36.2
High degree of independence	55.3*		47.8*		48.9
Opportunity to participate in nursing practice projects	47.5‡		20.0‡		24.2
Less non-nursing/administrative activities	31.2		36.9		36.1
Work-life-balance					
Good compatibility of work and family/leisure time	76.6*	Yes	82.3*	Yes	81.4
Reliable duty rosters	73.8‡		83.0‡		81.6
Prompt compensatory time after overtime hours/stressful shifts	59.2*		66.8*		65.6
Flexible adjustment of contractual working hours (full-time, part-time, sabbatical)	51.1*		57.6*		56.6
Possibility to waive night shifts	27.0		29.1		28.8

* p<0.05.

† p<0.01.

‡ p<0.001.

§ Nurses with 3-year vocational education from nursing schools.

BSN, Bachelor of Science in Nursing.

Findings on the importance of salary showed statistically significant differences for all items surveyed between the BSN and vocationally educated nurses. Mirroring the question on recruitment, 77.0% of BSN (compared with 46.6%, p<0.001) rated salary according to level of qualification as very important and one of the top three retention factors.

Regarding clinical roles, a higher proportion of BSN placed great importance on advanced tasks (59.9% vs 32.0%, p<0.001), having a high level of independence (55.3% vs 47.8%, p=0.020) and opportunities to participate in nursing practice projects (47.5% vs 20.0%, p<0.001). Reducing non-nursing or administrative activities was of less importance to BSN than to vocationally educated nurses, but non-significant.

Three work-life-balance factors were of significantly lower importance to BSN than vocationally educated nurses, including the compatibility of work with family or leisure time (76.6% vs 82.3%, p=0.024). The possibility to adjust contractual working hours or waive night shifts was also of less importance to BSN than to vocationally educated nurses, but differences were non-significant.

### Retention: qualitative findings

On retention, the qualitative results showed five key themes, namely (1) teamwork including BSN acceptance and role transparency; (2) engaging leadership towards BSN; (3) tailored career opportunities: supporting BSN to grow; (4) salary; as well as (5) BSN-specific expanded activities and tasks.

### Teamwork including BSN acceptance and role transparency

Good teamwork was mentioned by the BSN not only as essential for recruitment but also for retention. The BSN stated that successful teamwork would require acceptance and recognition of the BSN role by both intraprofessional and interprofessional teams. The visibility of BSN-roles within hospitals was also seen as crucial for receiving positive recognition and ensuring retention.

One BSN mentioned the need for proactively communicating her role within the team. At the same time, she actively involved colleagues.

But that’s simply because I handle my work very transparently. I involve [my colleagues] in my work. I inform them when I’ve done something, when I’ve finished something. And if you really value the opinion of others and ask for it, then it’s also very well regarded. But I think that’s always up to you. (ID6, BSN, Pos. 115–116)

In addition, several BSN highlighted the need of establishing hospital-wide BSN networks because BSN were often the minority on wards. Networks were seen as a way for BSN to connect beyond their wards and create a supportive team dynamic.

I would like to emphasise again… that I think it is very, very helpful when nurses with a background in nursing science understand themselves as a team in a way or a community feeling… have a place to exchange ideas with colleagues who are similarly interested… and dealing with nursing topics in a different way than is possible in day-to-day work in direct patient care. That you have this space for exchange… and that you also simply realise that ‘I’m not alone here…’. (ID21, BSN, Pos. 100)

The CNO and NSC/other did not distinguish between BSN and vocationally educated nurses when speaking about the need for supportive work environments and good teamwork as part of hospital retention strategies as they believed these factors were equally important for all nurses.

### Engaging leadership towards BSN

Most BSN articulated the need for an engaging leadership style which was described as appreciative, proactive, with regular dialogues initiated by supervisors and an ‘open door’ policy. Additionally, the receptiveness of managers to new ideas and concepts by BSN was seen as vital for retention in direct patient care.

I don’t know if you can force it, but by being open to new ideas and employees, because that’s the future: academic nursing. Otherwise, you lose people to management. It’s not bad either, you need people everywhere, but then people leave the bedside because there is still not enough of a concept [for BSN] at the bedside and not enough openness towards people [BSN] who wish to work at the bedside. (ID34, BSN, Pos. 93)

The majority of CNO and NSC/other considered leadership style as the most important retention element. NSC/other, in particular, often viewed themselves as a resource for BSN students and BSN staff, taking an advisory and supportive role. CNO and NSC/other recognised that more opportunities for BSN participation should be enabled by managers. Some interviewees stressed that engaging leadership also included a supportive corporate culture, higher appreciation of BSN by leaders as well as committed managers who understand that higher skill-mix is essential for improving patient care.

### Tailored career opportunities

Most BSN interviewees mentioned that retention requires providing individualised, tailored career development. Engagement from CNO for career support was seen as highly important. While one BSN highlighted a proactive culture where nurses are continually encouraged to develop professionally, another BSN expressed frustration with insufficient managerial initiative, noting that without active self-engagement, career progression seemed unlikely.

As far as I’m concerned, I would also change the fact that there isn’t enough communication from the leadership level with staff—about things like: ‘What are my competencies? Where are we heading?’ Because I feel that if you don’t take the initiative yourself, you just drift along and nothing more happens. You always have to act on your own. (ID34, BSN, Pos.79)

The CNO and NSC/other acknowledged that providing career opportunities with individual support and counselling were shown to foster trust and contribute to retention. Some also stressed that BSN should also take initiative and demonstrate their skills and competencies through practice initiatives or development projects.

### Salary

Five of the six BSN mentioned that a salary grade reflecting their higher qualification compared with vocationally educated nurses was crucial for both R&R. Higher pay was perceived as a demonstration of the employer’s recognition and appreciation of their expertise and qualification.

And I think that’s a bit of a shame, because then you don’t need to get a bachelor’s degree. Because, especially financially, you don’t get anything out of it and that frustrates me sometimes. So yes, very. (ID28, BSN, Pos. 18)

One BSN considered equal salary reasonable if no extra roles or responsibilities were assigned.

Most CNO and NSC/other acknowledged that increased salary reflects the value of the additional skills that BSN bring to the healthcare setting. However, they also clarified that in Germany, salary is typically linked to the tasks of the job and not to the qualification. Although some job profiles included additional, expanded tasks for BSN, salary was not always higher. The lack of a formal classification for BSN in the existing salary scales meant that BSN salary often depended on negotiations with the CNO on the actual job profiles and expanded scope of tasks.

One CNO referred to the large cross-hospital variability in the existence of different payment and incentive schemes and stressed that despite the existing tariff system, there are options available to pay BSN a higher salary if linked to BSN-specific roles or tasks:

There are monetary incentive systems, we pay academically qualified nursing staff differently if they have a specific task within the framework of the tariff system. …It is often said that in nursing there is no classification for bachelor’s and master’s degrees. Of course, there is, it is just a question of definition, whether a company wants to give the nursing staff a defined different task within nursing. (ID29, CNO, Pos. 48–49)

### BSN-specific expanded activities and tasks

Assuming expanded activities was highlighted as an important retention factor for BSN in clinical care. Full utilisation of their qualifications was considered as contributing significantly to job satisfaction. One BSN stressed that expanded roles in patient care are crucial to maintaining motivation. Otherwise, frustration may grow, eventually resulting in staff leaving the employer.

Especially regarding long-term retention, you need to be more committed, because people will become impatient at some point. If you’re always just seen as a nurse… and can’t do more with the bachelor’s degree, you get frustrated and then employees leave. And I believe that if you don’t offer specific, concrete things or measures or say, ‘hey, can you imagine this and that’, then staff become impatient, then they leave shift work and then they leave the hospital. (ID28, BSN, Pos. 50)

CNO and NSC/other agreed that BSN should be entrusted with expanded responsibilities and greater autonomy to fully leverage their competencies.

However, BSN frequently noted that staffing shortages and unstable rosters due to high sick leave and attrition rates often prevented the practical implementation of these planned activities. CNO and NSC/other confirmed these staffing shortages were a critical barrier. One NSC/other described how staffing shortage directly undermined the implementation of expanded BSN roles.

One of the main issues is the volume of work and the shortage of staff. We have a lot of colleagues who are really great and have great ideas, and how difficult it is to get them involved in this work… we need them for the night shift or for the early shift. So this is a fundamental issue for all conceptual work, because above all there is this huge supply pressure and staff shortage which doesn’t allow us to do such developments. (ID30, NSC/other, Pos. 72)

### Retention: triangulation

The survey and interview data reinforced that engaged leadership, tailored and proactive career opportunities as well as salary based on qualifications were decisive factors for retention. The interviews revealed that BSN placed a high value on the recognition and acceptance of their roles within teams and by CNO. The willingness of CNO to support BSNs’ ideas and their expanded clinical care tasks was considered crucial for retaining the interviewed BSN in direct patient care roles. Regarding workplace roles and responsibilities, BSN emphasised the importance of performing expanded tasks with a high degree of autonomy. However, in practice, staffing shortages often prevented BSN from working in expanded roles.

## Discussion

This study showed that on recruitment, BSN rated besides good teamwork, structured BSN-specific onboarding programmes and salary by qualification as highly important. Interviewees additionally emphasised the need for targeted job advertisements with BSN-specific profiles, mentorships for BSN, hospital’s external communication strategies and networking to improve recruitment. On retention, both survey and interview data pointed to the importance of engaging leadership, salary by qualification and tailored career opportunities for BSN. The interviews further highlighted the relevance of BSN-specific expanded clinical tasks and protected time for additional tasks. Across both dimensions, recruitment and retention, the importance of good teamwork, including BSN acceptance, role clarity and salary by qualification emerged as highly relevant.

Good teamwork was one of the top factors for both R&R in our study for BSN as well as vocationally educated nurses. Previous studies have shown that effective teamwork is valued by vocationally educated nurses^10^ and that positive working relationships and collegial support contribute to retention.^4 7^ In our findings, teamwork was equally important for both vocationally educated nurses and BSN. Recognition and appreciation by colleagues have previously been linked to higher job satisfaction among BSN,^26^ which aligns with our results. We found that acceptance of the BSN role is essential for effective intraprofessional and interprofessional collaboration. In addition, peer networking among BSN was seen as beneficial for the networking of BSN within a hospital.

Our findings illustrated that salary in relation to qualification was consistently rated higher by BSN. Most BSN expected their academic qualification to be financially recognised. Previous research has shown that salary is a relevant factor for employer attractiveness, staff retention and job satisfaction among nurses.^1 4 7 10 40^ The WHO emphasised the need for fair and effective remuneration to ensure the R&R of health and care workers.^41^ In Germany, pay structures for academically qualified nurses vary considerably, despite the existing tariff system.^31^ Research has shown that BSN reported high dissatisfaction with their salaries.^26^ Our study showed that some hospitals identified ways of paying BSN higher salaries, showing that there is a certain degree of flexibility, despite the primarily task-based salary tariff system in Germany. At the same time, the interviews showed the divergent and intransparent salary levels across and within some hospitals. This underlines the importance of revisiting BSN salary and payment structures in Germany.

Several BSN specific measures for recruitment emerged from the findings of our study.

First, while the importance of structured onboarding and mentorship programmes has been identified for all nurses in previous studies,^7 10^ the results from our study indicate that such programmes, including trainee programmes and access to designated mentors, are essential specifically for attracting BSN. Second, in our study, BSN-specific job advertisements were identified as a key factor for successful recruitment. However, previous research has shown that job advertisements often lack detail regarding descriptions of specific BSN roles.^42^ It remains unclear why only a few hospitals seem to have implemented this measure. Third, hospitals’ external communication was seen as important for attracting BSN. Our study showed that information about BSN recruitment was frequently neither publicly accessible nor easily identifiable by BSN searching for a new job. A previous international review across five countries also showed that applicants value information about nursing roles and opportunities. However, such details are often missing or insufficiently presented on hospital websites,^43^ suggesting that hospital management can improve R&R with a set of specific measures, most of which are low-cost and easy to implement.

With regard to retention, our findings have identified several measures that are specifically relevant for BSN. First, our results underscored the importance of strong and appreciative leadership for retaining BSN. Specifically, our study highlighted the pivotal role of the CNO in this context. Regular dialogue between the CNO and BSN was identified as critical, along with the wish that CNO should be open to BSN-initiated ideas and contributions. Several studies have shown that leadership is associated with nurse satisfaction, recruitment and retention and work environments.^4 6 44–46^ Effective nursing leadership involves fostering autonomy, flexibility and trust,^4 47^ while staff recognition and encouragement of initiatives were associated with higher satisfaction and retention.^4 45^ In addition, evidence emphasised the role of long-term strategies prioritising the integration of research into nursing practice.^48^ In line with this, BSN reported greater satisfaction when recognised by their supervisors,^26^ and regular communication with managers has been associated with improved retention.^4^ The WHO has underscored supportive management as a pillar for effective retention of the health and care workforce.^41^

Second, our findings have shown the high relevance of tailored career opportunities for BSN retention if supported by CNO engagement. Previous research has identified individualised career pathways as essential for retaining nurses.^1 4 7 41 47^ Conversely, a lack of opportunities for advancement has been associated with dissatisfaction, particularly among BSN.^26 40^

Third, our findings have indicated that BSN qualifications aligned with clinical roles and job content are crucial for retention. Many BSN reported the underutilisation of their competencies, and only 60% stated that they work in roles matching their qualifications. In addition, specialised BSN roles and tasks have been implemented unevenly across participating hospitals. These results mirror previous research showing that BSN roles are inconsistently defined and used across hospitals in Germany.^23 28^ While a previous study surveying 273 BSN showed that more than half reported satisfaction with their tasks, many expressed dissatisfaction about the inadequate use of their skills and knowledge.^26^ In light of expanding nursing roles in Europe, the WHO has called for continuous professional development across the career spans.^41^ Our findings, however, showed that many BSN often remain underused in practice, despite their strong motivation to take on expanded responsibilities.

This study has several strengths and limitations. A key strength is its mixed-methods design, to our knowledge the first of its kind on R&R measures by nursing qualification. Triangulating survey data with interviews from BSN, CNO and NSC/other provided insights unattainable by using either method alone. The quantitative sample focused on hospitals with a higher proportion of BSN and innovative R&R measures, highlighting practices in pioneer hospitals that may serve as examples for others. This indicates that the intended focus on innovative hospitals was achieved, as reflected in both the survey data and the interview findings, including the identification of specific R&R measures. Limitations include the non-representative hospital sample, which limits the generalisability of the findings, the small number of interviews with BSN and the absence of vocationally educated nurses among the interviewees. As BSN respondents were younger and less experienced, group differences may partly reflect career-stage rather than qualification alone and should be interpreted accordingly. Future research should explore R&R in relation to qualification and different life stages of nurses, such as early-career and mid-career nurses, older nurses and diverse ethnic and gender backgrounds. In addition, the response rate of 14.0% can be considered low and may partly be explained by survey fatigue, as many of the participating hospitals are actively involved in nursing research and educational activities. The possibility of non-response bias cannot be excluded, as no specific adjustments were applied. Another potential limitation is the deductive-inductive coding approach, which risked constraining qualitative findings to survey items; this was mitigated by extensive inductive coding. Despite these limitations, this is the first mixed-methods study in Germany as well as one of the largest quantitative studies on R&R internationally by nursing qualification in hospitals.

Our findings have important implications for both hospital management and policymakers. There is a need for more targeted R&R strategies that articulate a clear and specific profile for BSN. This includes the creation of job advertisements specifically for BSN, outlining career development opportunities, emphasising the BSN role within the clinical team and remuneration by qualification.

## Conclusion

Several R&R measures specific for BSN were identified, indicating that qualification-tailored strategies are needed. Clear BSN profiles, expanded clinical roles, protected time for additional tasks, tailored career opportunities and qualification-based salary structures were identified as highly important. Management and policy support can facilitate the successful integration and retention of BSN in hospitals.

## Supplementary material

10.1136/bmjopen-2025-113495 online supplemental file 1

## Data Availability

Data are available upon reasonable request.
