# Supplementary material for: Perceived importance of recruitment and retention measures for bachelor-qualified nurses in German hospitals: mixed-methods study
Source: BMJ Open. 2026 Aug 10;16(8):e113495. doi: 10.1136/bmjopen-2025-113495 (PMC13475144; doi:10.1136/bmjopen-2025-113495)
Supplement: Supplementary file 1 [file bmjopen-2025-113495.reviewer_comments.pdf]

PEER REVIEW HISTORY

BMJ Open publishes all reviews undertaken for accepted manuscripts. Reviewers are asked to complete a checklist review form and are provided with free text boxes to elaborate on their assessment. These free text comments are reproduced below.

ARTICLE DETAILS

Title (Provisional)

Perceived importance of recruitment and retention measures for bachelor qualified nurses in German hospitals: mixed-methods study

Authors

Maier, Claudia B. Bettina; Reidt, Charlotte; Köppen, Julia; Baumann, Mandy; Mazur, Ana

---

VERSION 1 - REVIEW

**Reviewer**	**1**
**Name**	**Axelsson, Malin**
**Affiliation**	**Malmö University, Department of Care Science, Faculty of Health and Society**
**Date**	**22-Dec-2025**
**COI**	**None**

Thank you for the invitation to review this manuscript.
The topic of the manuscript is relevant and interesting but there is no clear rational and no clear study aim which makes it difficult to assess the parts of the method and the method is not clearly described, which makes it difficult to interpret the results.

Title: Recruitment and retention: What factors are important for bachelor qualified nurses in German hospitals? Results from a mixed methods study (BSN4Hospital)

ID: bmjopen-2025-113495

The study has an interesting focus on recruitment and retention of registered nurses. My comments are outlined below.

Title: The abbreviation in the title is impossible to understand. What are the important factors directed towards and what is the outcome variable?

Abstract and manuscript: there is no clear aim of the study, which makes the study difficult to understand and raises concerns about the methods used.

Strengths and limitations: What is the definition of innovative hospitals? Sample size? Response Rate? Scientific basis of the survey and interview guides? Qualitative analysis?

Introduction: It may be a German situation but not international that low intake to nursing education is one explanation, which needs clarification. The following statement is unclear: "Challenges have been reported in identifying suitable areas of work in clinical practice and implementing organisational adjustments." Do not nurses with bachelor's degrees work in direct patient care to increase patient safety and improve nursing care based on scientific evidence? Is not the main issue, that there is a vocational nursing education? How many nurses are educated in respective nursing education? Is the vocational nursing education the largest? Why is there a vocational nursing education? Is this nursing education more like an education for nurse aides/assistant nurses i.e. not a university education? Are these nurses qualified as registered nurses? Are these two types of nurses comparable? What is the rationale for the study?

Method: Without a clear aim it is impossible to assess if the methods used (participants, setting, datacollection, analyses) are relevant? What is APN and why are these nurses mentioned but not included in the current study? How was informed consent obtained? On what scientific basis were the items and the interview guide developed? How were the items grouped in different constructs/dimensions? Why was a 4-point-likert scaled dicotomised based 1 alternative and 3 alternatives? Why were all these included in the interviews: BSN, APN, hospital managers and nurse scientists, nursing students? Which type of nursing students? Can the following really be possible after so few interviews with a scattered sample and before the analyses was done? "In the final three interviews, no new themes emerged of relevance to the research question, suggesting that data saturation was reached" Saturation is not the correct term based on the qualitative method used. Unclear what specific type of qualitative analysis that was used and how the analysis was conducted. How were the data from different professions handled in the analysis? The quotations are primarily from BSN even though they were the smallest group? Triangulation is not described. Which is the primary outcome? The statistical analyses describe do not match the information in the tables.

Results: Without a clear aim, it is difficult to interpret the findings and assess if the aim is answered. There is a repetition of results in the tables for instance Good team work. How can the same results be relevant for different aspects i.e. both retention and recruitment? It is not clear from the tables which analyses that were used.

Discussion and conclusion: The above needs to be taken into consideration in the discussion and conclusion of the results. Limitations need to be discussed in relation to methodological literature.

**Reviewer**	**2**
**Name**	**Lancia, Loreto**
**Affiliation**	**University of L'Aquila**
**Date**	**05-Mar-2026**
**COI**	**None**

This manuscript presents a well-designed convergent mixed-methods study examining recruitment and retention (R&R) strategies for bachelor-qualified nurses (BSN) in German hospitals. The topic is highly relevant given the current nursing workforce shortages and the ongoing academicisation of nursing across Europe. The mixed-methods design, the large quantitative sample, and the triangulation with qualitative interviews represent clear strengths. Overall, the manuscript is well structured and clearly written.

However, several clarifications and contextual additions would strengthen the methodological transparency and international interpretability of the findings.

First, the manuscript would benefit from a clearly stated aim at the end of the introduction. While the objectives are described, an explicit aim statement would help guide the reader.

Second, the introduction would benefit from a clearer description of the German nursing education and professional qualification system. For international readers, it would be helpful to briefly explain how the dual pathway (three-year vocational education vs. bachelor-level education) leads to professional qualification and how this model differs from many other European countries where nursing education is delivered exclusively through universities (e.g. Italy, Spain, Portugal, or the UK). Providing this context would help readers better understand the relatively low proportion of BSN in Germany and the relevance of recruitment and retention strategies targeting this group.

Third, the study sample consists of hospitals characterised by an above-average proportion of BSN and by the presence of innovative R&R strategies. While this approach is understandable for exploratory purposes, it introduces a potential selection bias because these organisations may differ substantially from typical German hospitals. The implications of this sampling strategy for the generalisability of the findings should therefore be discussed more explicitly.

Fourth, BSN respondents were significantly younger and had fewer years of professional experience compared with vocationally educated nurses. Some differences interpreted as qualification-related may therefore partly reflect career-stage effects. This potential confounding should at least be acknowledged in the discussion.

Fifth, the manuscript reports that Likert-scale responses were dichotomised into “very important” versus all other categories. While this approach simplifies the analysis, it reduces variability and may affect interpretation. A brief justification for this analytical choice would be helpful.

Finally, the overall survey response rate appears relatively low (13.9%). The possibility of non-response bias should therefore be discussed.

Minor issues:

• Line 164: replace “In Addition” with “In addition”.

• Table 3: replace “bevor signing” with “before signing”.

VERSION 1 - AUTHOR RESPONSE

Reviewer: 1
Dr. Malin Axelsson, Malmö University
Comments to the Author:
Thank you for the invitation to review this manuscript.
The topic of the manuscript is relevant and interesting but there is no clear rational and no clear study aim which makes it difficult to assess the parts of the method and the method is not clearly described, which makes it difficult to interpret the results.
Kind regards,
Malin Axelsson

Response by authors: We thank the reviewer for her time reviewing the manuscript and the constructive comments.

1. Title: The abbreviation in the title is impossible to understand. What are the important factors directed towards and what is the outcome variable?

Response by authors: Thank you. We have revised the title and removed the abbreviation.

2. Abstract and manuscript: there is no clear aim of the study, which makes the study difficult to understand and raises concerns about the methods used.

Response by authors: Thank you, this was also mentioned by reviewer 2. We have added an overall aim to the abstract and at the end of the introduction.

3. Strengths and limitations: What is the definition of innovative hospitals? Sample size? Response Rate? Scientific basis of the survey and interview guides? Qualitative analysis?

Response by authors: Thank you for this important comment. We agree that the definition of “innovative hospitals” requires further clarification. In this study, “innovation” was understood in relation to the German context, where the proportion of academically qualified nurses in acute care is still relatively low. Hospitals were therefore considered “innovative” if they demonstrated explicit measures or interventions to recruit, employ, or retain nurses with a bachelor’s degree (BSN), for example through organizational strategies or specific interventions as stated on the websites of the hospitals. We have expanded on this in the Methods sections.

In addition, we have also expanded the list of bullet points in the "Strengths and Limitations of This Study" section.

4. Introduction: It may be a German situation but not international that low intake to nursing education is one explanation, which needs clarification. The following statement is unclear: "Challenges have been reported in identifying suitable areas of work in clinical practice and implementing organisational adjustments." Do not nurses with bachelor's degrees work in direct patient care to increase patient safety and improve nursing care based on scientific evidence? Is not the main issue, that there is a vocational nursing education? How many nurses are educated in respective nursing education? Is the vocational nursing education the largest? Why is there a vocational nursing education? Is this nursing education more like an education for nurse aides/assistant nurses i.e. not a university education? Are these nurses qualified as registered nurses? Are these two types of nurses comparable? What is the rationale for the study?

Response by authors: Thank you for your comment. Reviewer 2 also pointed out some ambiguities in this regard. We have added comprehensive details about nursing education in Germany.

5. Method: Without a clear aim it is impossible to assess if the methods used (participants, setting, data collection, analyses) are relevant?

Response by authors: Thank you for your advice on methodology. We have added an overarching aim.

6. Method: What is APN and why are these nurses mentioned but not included in the current study?

Response by authors: We have removed the sentence on APN (Advanced Practice Nurses) as this group of nurses is distinctly different from BSN and has been excluded from this manuscript.

7. Method: How was informed consent obtained?

Response by authors: Thank you for this comment. We have clarified the informed consent procedures in the Methods section for both the survey and the interviews. For the survey, informed consent was obtained electronically prior to participation, and participants were required to provide consent before accessing the questionnaire. For the interviews, written informed consent was obtained prior to each interview.

8. Method: On what scientific basis were the items and the interview guide developed?

Response by authors: Thank you for this comment. Detailed information on the development of the survey items and interview guide is provided in the published study protocol (Ref. 33), which includes the full instruments. We have added a reference to the study protocol in the Methods section to improve transparency.

The interview guide was developed based on the existing literature on recruitment and retention of nurses and the current situation in Germany, as outlined in the study protocol (Ref. 33). In addition, selected survey items (e.g., regarding tasks and roles of nurses) informed the development of the interview questions to ensure alignment between the qualitative and quantitative components of the study. The guide was developed iteratively within the research team and refined through internal discussions.

9. Method: How were the items grouped in different constructs/dimensions? Why was a 4-point-likert scaled dicotomised based 1 alternative and 3 alternatives?

Response by authors: The original four-point response scale ("unimportant", "less important", "important", "very important") showed a strong skew towards high ratings. When combining the categories "important" and "very important", most items reached endorsement rates between 78% and >99%, indicating marked ceiling effects and limited discriminatory power between items. To allow for a more meaningful differentiation, analyses therefore focused on the category "very important", with the remaining response options grouped into a single comparison category. This approach enabled a clearer identification of aspects considered particularly salient by respondents and resulted in a wider and more informative distribution of values. Accordingly, the analyses presented are based on this dichotomization. We have clarified this recoding procedure and its rationale in the methods section.

10. Method: Why were all these included in the interviews: BSN, APN, hospital managers and nurse scientists, nursing students? Which type of nursing students?

Response by authors: The group of APN and nursing students has been removed because they are not relevant to this manuscript.

11. Method: Can the following really be possible after so few interviews with a scattered sample and before the analyses was done? "In the final three interviews, no new themes emerged of relevance to the research question, suggesting that data saturation was reached" Saturation is not the correct term based on the qualitative method used.

Response by authors: Thank you for this helpful comment. We agree that the term "data saturation" is not appropriate for the qualitative approach used in this study and that the original wording was misleading, particularly in suggesting that the absence of new themes could be determined prior to the completion of the analysis.

We have therefore removed this statement and revised the manuscript accordingly. Rather than referring to saturation, we now describe the dataset in terms of its adequacy for the analytic process. Specifically, we have relocated this consideration to the coding and analysis section and clarified that the dataset was judged to be sufficiently rich to support meaningful coding and the development of themes relevant to the research question.

12. Method: Unclear what specific type of qualitative analysis that was used and how the analysis was conducted. How were the data from different professions handled in the analysis?

Response by authors: Thank you for this comment. We have revised this section to more clearly specify the qualitative approach used and to provide a more detailed description of the analytic process. In particular, we now explicitly state that the analysis followed qualitative content analysis according to Kuckartz and describe the deductive–inductive development and iterative refinement of the category system. Moreover, we have clarified how data from different professional groups were handled in the analysis. Interviews from the selected groups (BSN, CNO, and nurse scientists/strategic roles) were analyzed as part of a single dataset, as all participants shared a common focus on academic nursing roles in hospital settings.

At the same time, differences in perspectives related to participants' professional roles were considered during the analysis. Rather than conducting a formal comparative analysis between groups, the aim was to capture a range of perspectives on recruitment and retention strategies across different roles within the same field. This has now been clarified in the Methods section.

13. Method: The quotations are primarily from BSN even though they were the smallest group?

Response by authors: Thank you for this comment. Interviews from all participant groups were included in the analysis and contributed to the development of the results. Quotations were selected to illustrate key themes and enhance clarity rather than to reflect the proportional distribution of the sample. Quotations from BSN were particularly useful for illustrating certain themes as they provided greater detail. We have clarified this in the Methods section.

14. Method: Triangulation is not described.

Response by authors: Thank you for this comment. We have added a description of the triangulation process to the Methods section. Specifically, we now clarify that a convergent mixed-methods approach with parallel analysis was used, in which quantitative and qualitative data were analysed independently and then systematically integrated through comparison using a triangulation matrix.

15. Method: Which is the primary outcome?

Response by authors: Thank you for this comment. This study was exploratory in nature and did not focus on a single primary outcome. Instead, two sets of outcomes (comprising 13 and 21 items, respectively) related to recruitment and retention were analyzed descriptively, and group differences between bachelor-qualified nurses and nurses with vocational education were analysed.

16. Method: The statistical analyses describe do not match the information in the tables.

Response by authors: Thank you for this comment. We revised the Methods section and the corresponding tables to ensure consistency in reporting. As age, years in the profession, and years in the current hospital were not normally distributed, these variables are now reported as median (Q1–Q3) instead of mean and standard deviation.

17. Results: Without a clear aim, it is difficult to interpret the findings and assess if the aim is answered. There is a repetition of results in the tables for instance Good team work. How can the same results be relevant for different aspects i.e. both retention and recruitment? It is not clear from the tables which analyses that were used.

Response by authors: Thank you for this useful comment. Some items (e.g., good teamwork, reliable duty rosters, or compatibility of work and family/leisure time) were considered relevant to both recruitment and retention, were based on the existing literature and were therefore included in both tables. However, these items were assessed separately for recruitment and retention, which is why the corresponding percentages differ between tables. We have clarified this in the methods section.

18. Discussion and conclusion: The above needs to be taken into consideration in the discussion and conclusion of the results. Limitation: need to be discussed in relation to methodological literature.

Response by authors: Thank you. Yes, indeed, we took account of the points raised and revised the text as appropriate. For instance, we have made considerable changes to the limitation section, to take account of the generally low response rate, the dichotomous analysis of the quantitative data, the low number of BSN interviewed, among others.

Reviewer: 2
Dr. Loreto Lancia, University of L'Aquila
Comments to the Author:
This manuscript presents a well-designed convergent mixed-methods study examining recruitment and retention (R&R) strategies for bachelor-qualified nurses (BSN) in German hospitals. The topic is highly relevant given the current nursing workforce shortages and the ongoing academicisation of nursing across Europe. The mixed-methods design, the large quantitative sample, and the triangulation with qualitative interviews represent clear strengths. Overall, the manuscript is well structured and clearly written. However, several clarifications and contextual additions would strengthen the methodological transparency and international interpretability of the findings.

<u>*Response by authors:*</u> We thank the reviewer for reviewing the paper and highlighting the strengths of the study as well as areas for improvements. We provide a one-by-one response to the comments, kindly see below.

1. First, the manuscript would benefit from a clearly stated aim at the end of the introduction. While the objectives are described, an explicit aim statement would help guide the reader.

<u>*Response by authors:*</u> Thank you, this was also mentioned by reviewer 1. We have added an overall aim to the abstract and at the end of the introduction.

2. Second, the introduction would benefit from a clearer description of the German nursing education and professional qualification system. For international readers, it would be helpful to briefly explain how the dual pathway (three-year vocational education vs. bachelor-level education) leads to professional qualification and how this model differs from many other European countries where nursing education is delivered exclusively through universities (e.g. Italy, Spain, Portugal, or the UK). Providing this context would help readers better understand the relatively low proportion of BSN in Germany and the relevance of recruitment and retention strategies targeting this group.

<u>*Response by authors:*</u> Thank you. This was also mentioned by Reviewer 1. We have added comprehensive details about nursing education in Germany.

3. Third, the study sample consists of hospitals characterised by an above-average proportion of BSN and by the presence of innovative R&R strategies. While this approach is understandable for exploratory purposes, it introduces a potential selection bias because these organisations may differ substantially from typical German hospitals. The implications of this sampling strategy for the generalisability of the findings should therefore be discussed more explicitly.

<u>*Response by authors:*</u> Thank you, indeed, the methodology was designed to identify hospitals with higher-than-average proportions of BSN and innovative R&R strategies. We had already mentioned that the study is not representative of all hospitals in Germany, but we expanded the section and made it more explicit so it is clearer that generalizability is limited.

4. Fourth, BSN respondents were significantly younger and had fewer years of professional experience compared with vocationally educated nurses. Some differences interpreted as

qualification-related may therefore partly reflect career-stage effects. This potential confounding should at least be acknowledged in the discussion.

Response by authors: Yes, thank you for raising this relevant point. Indeed, this reflects (at least partly) the still young existence of BSN educational programmes in Germany. We have added a sentence in the discussion on potential confounding and elaborated on it.

5. Fifth, the manuscript reports that Likert-scale responses were dichotomised into "very important" versus all other categories. While this approach simplifies the analysis, it reduces variability and may affect interpretation. A brief justification for this analytical choice would be helpful.

Response by authors: Thank you. Reviewer 1 also mentioned that. The original four-point response scale ("unimportant", "less important", "important", "very important") showed a strong skew towards high ratings. When combining the categories "important" and "very important", most items reached endorsement rates between 78% and >99%, indicating marked ceiling effects and limited discriminatory power between items. To allow for a more meaningful differentiation, analyses therefore focused on the category "very important", with the remaining response options grouped into a single comparison category. This approach enabled a clearer identification of aspects considered particularly salient by respondents and resulted in a wider and more informative distribution of values. Accordingly, the analyses presented are based on this dichotomization. We have clarified this recoding procedure and its rationale in the methods section.

6. Finally, the overall survey response rate appears relatively low (13.9%). The possibility of non-response bias should therefore be discussed.

Response by authors: Thank you for this important comment. We have clarified the reporting and interpretation of the response rate in the revised manuscript. The overall response rate across all participants was approximately 20%. However, analyses were restricted to complete cases, resulting in a response rate of 14.0%. We have also added a brief discussion of this aspect in the limitations section. The response rate can be considered low and may partly be explained by survey fatigue, as participating hospitals are actively involved in nursing research and educational activities. In addition, the possibility of non-response bias cannot be excluded, as no specific adjustments (e.g., weighting) were applied. Kindly note: We had originally obtained a response rate of 13.9%, but after recalculation and taking account of rounding errors, we arrived at 14% and have corrected this in the revised manuscript.

7. Minor issues:
• Line 164: replace "In Addition" with "In addition".
• Table 3: replace "bevor signing" with "before signing".

Response by authors: Thank you, this is corrected. We have fixed that.
